# Evaluation of three PCR-based diagnostic assays for detecting mixed *Plasmodium *infection

**DOI:** 10.1186/1756-0500-3-88

**Published:** 2010-03-31

**Authors:** Tonya Mixson-Hayden, Naomi W Lucchi, Venkatachalam Udhayakumar

**Affiliations:** 1 Malaria Branch, Division of Parasitic Diseases, National Center for Zoonotic Vector-Borne and Enteric Diseases, Coordinating Center for Infectious Diseases, Centers for Disease Control and Prevention, Atlanta, GA, USA; 2 Atlanta Research and Education Foundation, Decatur GA, USA

## Abstract

**Background:**

One of the most commonly used molecular test for malaria diagnosis is the polymerase chain reaction (PCR)-based amplification of the 18S ribosomal DNA (rDNA) gene. Published diagnostic assays based on the 18S gene include the "gold standard" nested assay, semi-nested multiplex assay, and one tube multiplex assay. To our knowledge, no one has reported whether the two multiplex methods are better at detecting mixed *Plasmodium *infections compared to the nested assay using known quantities of DNA in experimentally mixed cocktails.

**Findings:**

Here we evaluated three PCR assays (nested, semi-nested multiplex, and one-tube multiplex) for the simultaneous detection of human malaria parasites using experimentally mixed cocktails of known quantities of laboratory derived DNA. All three assays detected individual species with high sensitivity and specificity when DNA was from any one single species; however, experimentally mixed DNA cocktails with all four species present were correctly identified most consistently with the nested method. The other two methods failed to consistently identify all four species correctly, especially at lower concentrations of DNA -subclinical levels of malaria (DNA equivalent to or less than 10 parasites per microliter).

**Conclusions:**

The nested PCR method remains the method of choice for the detection of mixed malaria infections and especially of sub-clinical infections. Further optimization and/or new molecular gene targets may improve the success rate of detecting multiple parasite species simultaneously using traditional PCR assays.

## Findings

Approximately 2 billion people are exposed to malaria with an estimated 250 million clinical cases and about 800,000 deaths annually [1,2]. Four Plasmodium species are known to cause malaria in humans: *P. falciparum, P. vivax, P. malariae *and *P. ovale*. Many malaria endemic regions report mixed infections of these species and the prevalence of mixed infections varies depending on the geographic region. For example, within India, Hamer et al. [3] reported 9.3% of malaria cases as mixed *P. falciparum*/*P. vivax *infection and Rasheed et al. [4] reported 18% of cases were *P. falciparum*/*P. vivax *mixed infection. The clinical presentation of malaria is often nonspecific; consequently many febrile illnesses with unknown etiology are attributed to malaria (resulting in presumptive diagnosis and treatment). Therefore, appropriate laboratory tools are critical for the prompt diagnosis and treatment of malaria. This is especially true for mixed infections which are often not recognized, and as a result, have been underestimated (reviewed in [5] and [6]).

Existing tools for the diagnosis of malaria include microscopy, rapid diagnosis tests (RDTs) and molecular tools (reviewed in [7]). For over a century, microscopy has remained the standard for malaria detection and species determination in many endemic areas. It is relatively inexpensive and allows for the quantification of parasitemia. However, several challenges exist in performing microscopic diagnosis for routine clinical use, especially in malaria endemic countries, including limited availability of well trained microscopists, lack of infrastructure (such as power supply) and training, and the possibility of misdiagnosis due to low parasitemia or mixed infections, even with experienced microscopists [8,9]. Furthermore, microscopy may not consistently detect all infecting species accurately [10]. For example, *Plasmodium knowlesi *(a monkey malaria recently discovered to also infect humans) has been falsely diagnosed as *Plasmodium malariae *using routine microscopy, and only through the use of a molecular method was this species correctly identified in humans [11].

RDTs are immunochromatographic tests designed to detect parasite products in human blood. Currently, the two most common RDTs use histidine rich protein-2 (HRP-2) or lactose dehydrogenase (LDH) as target proteins for detection [12]. RDTs are increasingly being used for malaria diagnosis because they are rapid and easier to use especially in resource limited settings. Good quality RDTs are generally as sensitive as microscopy in detecting *P. falciparum *(about 100 parasites per microliter) but they are much less sensitive in identifying other species of malaria parasites. Because RDTs are only qualitative tests, the density of parasitemia cannot be accurately determined. Additionally, RDTs can result in false positives since antigen persists up to one month after the clearance of parasites.

Molecular methods are proving to be useful in species identification and accurate detection of mixed species infections in addition to the detection of subclinical infections [13]. As we move towards malaria elimination/eradication phases, tools to detect sub-clinical levels of infections will aid in malaria control programs. However, the use of molecular tools is hampered by some factors, including high cost of initial equipment setup and the inability to obtain reagents due to infrastructure in many field settings. Additionally traditional molecular methods can be labor intensive and may be prone to amplicon contamination thus requiring advanced technical knowledge. The most commonly used molecular test for malaria diagnosis is the polymerase chain reaction (PCR) -based amplification of the 18S ribosomal DNA (rDNA) gene which allows detection of the different species of human malaria parasites based on different sized PCR products [14-21]. The nested PCR method developed by Snounou et al [20] has been widely used in laboratory studies and in clinical diagnosis, including in a reference diagnostic laboratory in the United States [13,15,17,22]. However, this method is time consuming, expensive, and labor intensive as it requires five separate PCR reactions to detect *P. falciparum*, *P. vivax*, *P. ovale*, and *P. malariae*. Sequence variation in strains of *P. ovale *have recently been shown to affect the ability of the nested Snounou primers to identify variants of *P. ovale*, leading to the development of new primers for this species in the nested PCR assay [23]. Alternative multiplex approaches have been developed including a semi-nested multiplex [18] and single round multiplex PCR [16] in an effort to simplify this method, both of which target a region of the 18S gene thus far not shown to vary within individual species specific strains of Plasmodium [23]. The semi-nested multiplex PCR [18] utilizes a universal 18S rDNA reverse primer and two forward 18S rDNA primers, one specific to the *Plasmodium *genus and one specific to all mammalian 18S rDNA. The primary *Plasmodium *genus specific forward primer and four species specific reverse primers are used in the secondary reaction. The authors claim that the sensitivity of the semi-nested multiplex is equivalent to detecting 0.1 parasites/μl (p/μl) in an experimentally mixed *P. falciparum/P. malariae *DNA cocktail, and indicate that the method confirmed microscopically identified coinfections in field samples. The single round multiplex [16] utilizes a *Plasmodium *genus specific reverse primer with four forward species specific primers. Padley et al. [16] claim the sensitivity of the assay to be 0.02 and 0.004 p/μl for *P. falciparum *and *P. ovale*, respectively, and did not test sensitivity of the assay for *P. vivax *and *P. malariae*. Additionally they claim the assay was able to detect all four species in simulated mixed coinfections; however the data is only shown for *P. vivax/P. falciparum *coinfections. In addition to traditional PCR-based molecular tools, real-time PCR has recently been shown to be a robust alternative for malaria diagnosis. However, several factors inhibit the use of this method in malaria endemic regions including cost, lack of infrastructure, and lack of technical support due to infrastructure problems. Additionally, false negatives for Plasmodium species remain a problem with the current real time assays due to sequence variation and competitive inhibition [24].

The role of molecular tools for the detection of malaria parasites, and especially the detection of mixed infections, is becoming clear [25] and reviewed in [5]. The three previously described PCR-based methods (nested, semi-nested, and single-tube multiplex) are all good alternatives to microscopy and RDTs. However, to our knowledge, no one has evaluated which of these traditional PCR methods is truly better at detecting mixed *Plasmodium *infections; therefore, we compared the two multiplex methods to the nested method using known quantities of laboratory derived *Plasmodium *DNA alone and in experimentally mixed cocktails containing all four species of DNA.

## Methods

### Parasite culture and DNA extraction

DNA was extracted from laboratory cultured *P. falciparum *(3D7) and from monkey derived *P. vivax *(SV4), *P. malariae *(Uganda I), and *P. ovale *(Nigeria I) using a QIAamp DNA Mini Kit (QIAGEN, Valencia, CA), according to the recommendations by the manufacturer. Thin smears were prepared, stained, and counted for each of the species to determine the percentage parasitemia. The total number of red blood cells (RBCs) per microliter was then determined for each species using a coulter counter and the number of malaria infected RBCs per microliter (p/uL) was calculated using the formula below:

DNA was isolated from a total of 8 × 10^6 ^parasites for each species and suspended in 200 μl of sterile TE; therefore, 1 μl of sample contained the DNA equivalent of 40,000 parasites. The stock DNA was aliquoted and stored at -20°C, from which seven 10-fold serial dilutions were prepared up to a final concentration equivalent of 0.04 parasite genomes/μl. Assay limits of detection of individual *Plasmodium *species and detection of multiple species from mixed DNA cocktails were evaluated over seven replicates. Experimentally mixed cocktails were made using two μl each of parasite DNA of varying initial concentrations (Table 1). One microliter of the mixture was then used in a 20 μl PCR reaction, equivalent to the final parasitemias of each species given in Additional file 1. Because the field of mixed infections is one that has received little attention (mainly due to diagnosis issues), the DNA ratios for the mixed cocktails were chosen to both, represent low levels of infection, which are commonly missed by microscopy [5,6,25,26], and to simulate varying combinations of mixed infections. In general, equimolar amounts of each species rarely occur in the field, and it is common for one species in the mixed infections to predominate (reviewed in [25]).

**Table 1:** Preparation of mock mixed infections

Well #	*P. falciparum*	*P. malariae*	*P. ovale*	*P. vivax*
	**Concentration in Parasites/μL**			

1	400	40	400	40

2	40	40	400	40

3	40	40	40	40

4	40	40	4	40

5	4	40	4	40

6	4	40	4	4

7	40	400	40	40

8	4	400	4	40

9	400	40	400	400

10	40	40	4	400

11	40	4	4	40

12	400	40	40	400

13 (F)	400	-	-	-

14 (M)	-	400	-	-

15 (O)	-	-	400	-

16 (V)	-	-	-	400

17 (N)	-	-	-	-

Mock mixed infections were prepared as shown. 2 ul of each species was mixed with equal volumes of the other species as shown. One microliter of each mixture was then used for the PCR assay.

All PCR assays were completed on a BioRad iCycler (BioRad, Hercules, CA). Nested PCR was performed with primers and cycling conditions described by Snounou et al. [20] with modifications described in [27] in a 20 μl reaction containing 1× buffer, 2.5 mM MgCl2, 200 μM dNTPs, 200 nM primers, and 1.25 units of Taq Polymerase (New England Biolabs, Ipswich, MA). The multiplex semi-nested and multiplex single round PCRs were performed in 20 μl reaction using Promega Taq PCR Master Mix (Promega, Madison, WI), per manufacturer's instructions. Multiplex semi-nested PCR was performed with primers described by Rubio et al. [18], modifying the annealing temperatures to 55°C for the primary reaction and 58°C for the nested reaction. The multiplex single round PCR was performed using primers and cycling conditions described by Padley et al. [16] with the addition of an extension step for one minute at 72°C. No template control (water) was run in each experiment. Five microliters (5 μl) of PCR product were visualized in 2% agarose gel stained with ethidium bromide.

## Results and Discussion

**Figure 1:**
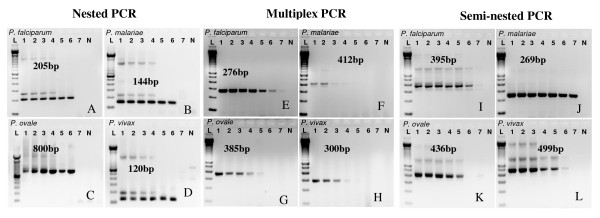
**Limits of detection of the three different methods at detecting the four Plasmodium species**. 10-fold serial dilutions of all four species were prepared starting from 40,000p/μl stock. Lane 1 = 40, 000p/μL, 2 = 4,000p/μL, 3 = 400p/μL, 4 = 40p/μL, 5 = 4p/μL 6 = 0.4p/μL and 7 = 0.04p/μL. One microliter of each of these dilutions was amplified as per the different protocols. Representative gels are shown for the nested (Snounou) (A-D), multiplex (Padley) (E-H), and semi-nested (Rubio) (I-L) results. The nested PCR was able to amplify up to 0.4p/μL for each of the species. The multiplex method amplified up to 0.4 p/μL for *P. falciparum*, 4 p/μL of *P. ovale *and 40p/μL of both *P. malariae *and *P. vivax *while the semi-nested PCR amplified up to 0.04p/μL for *P. malariae*, 0.4p/μL for both *P. falciparum *and *P. vivax *and 4p/μL for *P. ovale*. L: 100 bp molecular weight marker.

The nested PCR assay was able to detect single *Plasmodium *species infections down to 0.4 p/μl (Figure 1A-D). The semi-nested multiplex assay was as sensitive as the nested assay at detecting *P. falciparum *and *P. vivax*, more sensitive at detecting *P. malariae *(0.04p/μL), and was only able to detect down to 4 p/μL for *P. ovale *(Figure 1I-L). The limit of detection for the multiplex single round protocol was comparable to the other protocols for *P. falciparum*, but was only able to detect down to 4 p/μl for *P. ovale *and 40 p/μl for *P. malariae *and *P. vivax*, a 100-fold difference in sensitivity (Figure 1E-H). Our levels of detection for *P. falciparum *in a singly infected sample are comparable to those reported by Rubio et al. [18] (0.1 p/μl), while sensitivity was greater than reported by Rubio et al. [18] for *P. malariae *(0.1 p/μl), and much less sensitive than reported by Padley [16] for *P. falciparum *(0.02 p/μl) and *P. ovale *(0.004 p/μl). Because we had to rely on parasite counts to determine our initial concentrations for the species for which we are unable to culture (i.e. *P. vivax*, *P. ovale*, and *P. malariae*), equal p/μl may not contain equal amounts of DNA due to the presence of schizonts, thus affecting our ability to replicate the sensitivity results previously reported by these authors.

**Figure 2:**
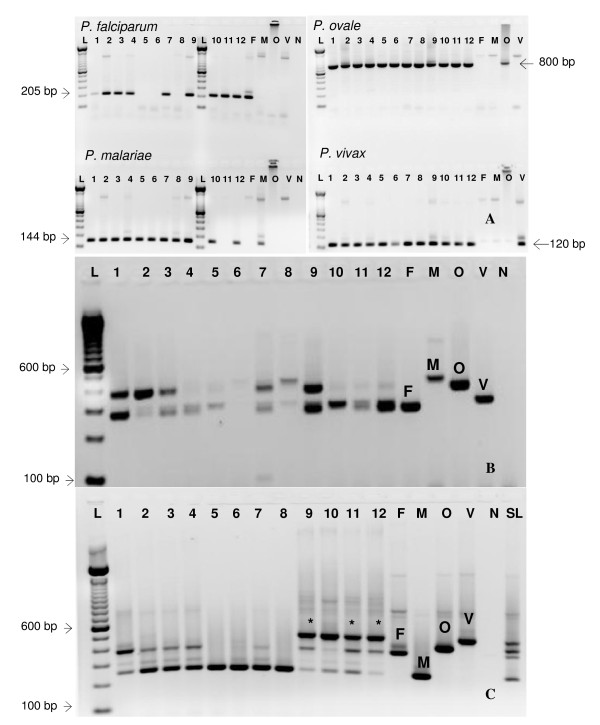
**Detection of mock mixed infections using the three different methods**. Mock mixed infections were prepared as per Table 1. One microliter of each of the mock mixed infection was amplified according to the different protocols resulting in the final parasite concentrations given by Additional file 1. Representative gels are shown for the nested (Snounou) (A), multiplex (Padley) (B), and semi-nested (Rubio) (C). Lane numbers correspond to the lanes as shown in Additional file 1. F: *P. falciparum*, M: *P. malariae*, O: *P. ovale*, V: *P. vivax*, L: 100 bp molecular weight marker, SL: Species Ladder created by mixing equal amounts of individually amplified species DNA using Rubio primers.

The accuracy of each method to detect all four human *Plasmodium *species in experimentally mixed infections was assessed over seven replicates. Only the nested PCR assay consistently detected all four species in the cocktails at the sensitivity levels reported for singly infected samples (Figure 2A, Additional file 1). However, the assay is more labor intensive than the other two methods, requiring five sets of PCR reactions for systematic detection of all four parasites.

The semi-nested multiplex method detected at best, three of the four species simultaneously (*P. falciparum *and *P. vivax *and either *P. malariae or P. ovale*, depending on concentration) as shown in Figure 2C (*) to as low as 10 p/μl. The assay was able to detect parasite concentrations as low as one p/μl in certain mixing schemes, in agreement with our level of sensitivity found for this assay for singly infected samples. In the mixed DNA cocktails, the semi-nested PCR assay was less sensitive in amplifying *P. falciparum *and *P. vivax *consistently (Figure 2 and Additional file 1). This method was successful in amplifying *P. falciparum *DNA 4/7 times in any of the cocktail combinations tested. The assay performed slightly better with *P. vivax*, correctly identifying the species 6/7 times when parasitemia was 100 p/μl. At lower parasitemias, the ability of the semi-nested assay seemed to depend on the proportion of *P. vivax *to *P. ovale*. For instance, when *P. vivax *and *P. ovale *were present in the mixture at 10 p/μl each, the assay correctly identified *P. vivax *4/7 times (Additional file 1), but 5/7 or 6/7 times if *P. ovale *was present at one p/μl. It appears that the performance of this method in amplifying *P. vivax *DNA was negatively affected whenever the concentration of *P. ovale *was higher than *P. vivax *(as noted in the failure of this assay to detect *P. vivax *when the ratio of *P. ovale *DNA to *P. vivax *DNA was 100:10 p/μl, Additional file 1). The assay performed quite well in amplifying *P. malariae *and *P. ovale *in mixed DNA cocktails, generally detecting these species greater than 50% of the time (Table 1). Occasionally, we were able to amplify all the four species but this was not consistent (2/7 times this method amplified all four species for the mixed DNA cocktail containing 10, 10, 1, 10 p/μl and 1/7 times in the cocktail containing 100, 10, 10, 100 p/μl of *P. falciparum, P. malariae, P. ovale, and P. vivax*). We had better success at amplifying three species simultaneously as shown in Figure 2C (*). Because the assay performs well for samples infected with only one species of *Plasmodium*, it is likely that competition for primers contributed to the inability to amplify all four species of *Plasmodium *simultaneously; therefore, in instances where multiple species of *Plasmodium *are suspected, the method can be run with only individual species specific primers to increase the specificity of the assay.

The one tube multiplex was the least sensitive, detecting at most only two species simultaneously in the mock mixed infections (Figure 2B). The assay correctly identified *P. falciparum *most consistently in the mixed DNA cocktails; however, it was especially poor at detecting *P. malariae *and *P. vivax*, correctly identifying these species less than 50% of the time (Additional file 1). This method performed reasonably well in identifying *P. ovale*, especially when the concentration of this DNA was equal to or greater than 10 p/μl, in agreement with the level of sensitivity found in the singly infected samples. We were able to detect 1 p/uL of *P. ovale *in the mixed mock infections using the semi-nested assay 22/42 times and the single tube multiplex 7/42 times, which is within an order of magnitude of the level of detection we found for single infections (4 p/uL). Occasionally, we were able to detect a lower than expected concentration of parasites in the mock mixed infections for multiplex single tube assays for *P. vivax *(less than 40 p/uL, 9/54 times), which was most probably by chance only. Competition between the primer pairs likely contributed to the inconsistencies of the semi-nested multiplex and single tube multiplex methods to correctly identify multiply infected samples.

Cost of each assay was calculated and included the price of reagents and plastic consumables (tips, tubes, etc.) for a single sample. The nested method was the most expensive (8 USD/sample), followed by the semi-nested method (5 USD/sample), and single tube multiplex (4 USD/sample).

Clearly the nested PCR method is the best PCR-based assay for the diagnosis of mixed infections and of subclinical infections among the three methods tested in this study. However, researchers should be aware of the sequence variability within strains of *P. ovale*, and thus utilize the primers for this species as described in [23]. Many of the inhabitants of high malaria endemic regions may harbor high levels of malaria parasites with no clinical symptoms (subclinical/asymptomatic infections) mainly due to acquired partial immunity. These infections are commonly missed by microscopy and RDT which usually detect 50-100 p/uL at best. As we move towards the malaria elimination/eradication phases suggested by the malaria community, subclinical infections will be common due to an increase in intervention programs; therefore, tools to detect sub-clinical levels of infections will be critical to determine the success of malaria control programs. Only molecular tools have been shown to accurately detect subclinical levels of infections. In this study, the nested PCR method, though tedious in nature, was the only method that consistently and accurately detected these levels. In addition, mixed infections have often been unrecognized or underreported [5,6,25,26] mainly due to limited detection tools [25]. Indeed, the use of molecular tools for malaria diagnosis has revealed that low-level mixed infections are common [26]. Further optimization of primers and reaction conditions could possibly improve the semi-nested and multiplex methods. This study suggests that diagnostic assays need to be given careful consideration before use in clinical diagnostic settings due to the probability of a high number of false negatives if these two multiplex methods are used. In areas where there are few species circulating, the semi-nested assay may be an acceptable assay; however, in areas where many species are circulating, the nested PCR assay would be the most appropriate diagnostic assay of these three. Alternative molecular methods using real time PCR [28,29] and loop mediated isothermal amplification (LAMP) [30-32] assays have been reported and further evaluation of these newer methods will be necessary to determine their utility for malaria diagnosis in the field. It is important to note that in at least one real time PCR 18S assay, it has been shown that if there is a greater than 10 fold difference in the parasitemia in a mixed infection, the species with the greater concentration of DNA will be the only species detected [24]. In addition, no reports have been made, to date, of multiplex-LAMP assays. Further research to develop simpler and field-usable molecular tools will enhance prompt and accurate diagnosis of malaria in endemic regions. The release of the whole genomes of *P. falciparum *and *P. vivax *should enable the identification of potential unique species-specific molecular targets to overcome some of the limitations associated with the existing multiplex molecular tools.

## Competing interests

The authors declare that they have no competing interests.

## Authors' contributions

TMH and NWL carried out the molecular assays and drafted the manuscript. VU conceived of and designed the study and helped draft the manuscript. All authors have read and approved the final manuscript.

## Supplementary Material

Additional file 1 **The success rate of each assay in detecting *P. falciparum*, *P. malariae*, *P. ovale*, and *P. vivax *at the varying parasite concentration within mock-mixed infections**. The nested and multiplex semi-nested PCR assay was replicated seven times for each of the mock-mixed infections, while only six replications were considered for the multiplex single round due to lack of amplification of the positive controls in one replication. Click here for file
