# Supplementary material for: Evaluation of three PCR-based diagnostic assays for detecting mixed Plasmodium infection
Source: BMC Res Notes. 2010 Mar 31;3:88. doi: 10.1186/1756-0500-3-88 (PMC2853551; doi:10.1186/1756-0500-3-88)
Supplement: Additional file 1 — The success rate of each assay in detecting P. falciparum, P. malariae, P. ovale, and P. vivax at the varying parasite concentration within mock-mixed infections. The nested and multiplex semi-nested PCR assay was replicated seven times for each of the mock-mixed infections, while only six replications were considered for the multiplex single round due to lack of amplification of the positive controls in one replication. [file 1756-0500-3-88-S1.DOC]

**Supplementary Table 1. The success rate of each assay in detecting *P. falciparum*, *P. malariae*, *P. ovale*, and *P. vivax* at the varying parasite concentration within mock-mixed infections.**

The nested and multiplex semi-nested PCR assay was replicated seven times for each of the mock-mixed infections, while only six replications were considered for the multiplex single round due to lack of amplification of the positive controls in one replication.

|  | # Parasites/ µl F/M/O/V |  |  |  |  |  |  |  |  |  |  |  |  |
| --- | --- | --- | --- | --- | --- | --- | --- | --- | --- | --- | --- | --- | --- |
| Assay |  | 100/10/  100/10 | 10/10/  100/10 | 10/10/  10/10 | 10/10/  1/10 | 1/10/  1/10 | 1/10/  1/1 | 10/100/  10/10 | 1/100/  1/10 | 100/10/  100/100 | 10/10/  1/100 | 10/1/  1/10 | 100/10/  10/100 |
| Snounou | F | 7/7 | 6/7 | 7/7 | 6/7 | 4/7 | 2/7 | 7/7 | 3/7 | 7/7 | 5/7 | 6/7 | 7/7 |
| (Nested) | M | 6/7 | 6/7 | 5/7 | 7/7 | 7/7 | 7/7 | 7/7 | 7/7 | 6/7 | 5/7 | 2/7 | 4/7 |
|  | O | 7/7 | 6/7 | 6/7 | 4/7 | 3/7 | 4/7 | 6/7 | 6/7 | 6/7 | 4/7 | 2/7 | 6/7 |
|  | V | 7/7 | 6/7 | 6/7 | 6/7 | 6/7 | 6/7 | 6/7 | 6/7 | 7/7 | 7/7 | 6/7 | 7/7 |
| Rubio | F | 2/7 | 2/7 | 4/7 | 4/7 | 3/7 | 4/7 | 4/7 | 2/7 | 3/7 | 2/7 | 3/7 | 4/7 |
| (Multiplex Semi-Nested) | M | 0/7 | 0/7 | 7/7 | 7/7 | 6/7 | 4/7 | 7/7 | 6/7 | 1/7 | 3/7 | 4/7 | 5/7 |
|  | O | 7/7 | 7/7 | 7/7 | 7/7 | 6/7 | 4/7 | 6/7 | 1/7 | 6/7 | 1/7 | 3/7 | 6/7 |
|  | V | 0/7 | 0/7 | 4/7 | 6/7 | 5/7 | 0/7 | 4/7 | 5/7 | 6/7 | 6/7 | 6/7 | 6/7 |
| Padley | F | 6/6 | 6/6 | 6/6 | 6/6 | 4/6 | 4/6 | 3/6 | 1/6 | 5/6 | 4/6 | 4/6 | 5/6 |
| (Multiplex Single Round) | M | 0/6 | 0/6 | 0/6 | 0/6 | 0/6 | 0/6 | 4/6 | 5/6 | 0/6 | 0/6 | 0/6 | 0/6 |
|  | O | 6/6 | 5/6 | 5/6 | 1/6 | 1/6 | 4/6 | 3/6 | 0/6 | 4/6 | 0/6 | 1/6 | 3/6 |
|  | V | 1/6 | 1/6 | 1/6 | 1/6 | 1/6 | 0/6 | 1/6 | 3/6 | 2/6 | 3/6 | 0/6 | 1/6 |
